# Predicting Preeclampsia Pregnancy Termination Time Using sFlt-1

**DOI:** 10.3389/fmed.2022.900639

**Published:** 2022-06-20

**Authors:** Hiroaki Tanaka, Kayo Tanaka, Sho Takakura, Naosuke Enomoto, Tomoaki Ikeda

**Affiliations:** Department of Obstetrics and Gynecology, Mie University School of Medicine, Tsu, Japan

**Keywords:** preeclampsia, sFlt-1, PlGF, prediction, Angiogenetic factors

## Abstract

**Background:**

The aim of this study was to determine the usefulness of placental growth factor (PlGF) and soluble fms-like tyrosine kinase-1 (sFlt-1) in predicting the time for pregnancy termination in pregnant women with known preeclampsia (PE) onset.

**Methods:**

Forty-four pregnant women diagnosed with PE (22 weeks 0 days to 33 weeks 6 days gestation) were included in this study. The levels of sFlt-1 and PlGF, and the sFlt-1/PlGF ratio were compared between the women that delivered in <24 h (T group) and those that delivered in more than 24 h (P group), and between women that delivered in <1 week (T group) and those that delivered in more than 1 week (P group). Cutoff values were calculated for the three markers that were the most significantly correlated with predicting pregnancy termination at <24 h and <1 week.

**Results:**

Among sFlt-1, PlGF, and sFlt-1/PlGF, sFlt-1 was the most significantly associated with the timing of pregnancy termination. sFlt-1 cutoff values of 8682.1 pg/ml (AUC 0.71; 95%Cl, 0.5191–0.9052) and 7,394.5 pg/ml (AUC 0.78; 0.78, 95%Cl, 0.6394–0.9206) for delivery in <24 h and delivery within 1 week, respectively, were important predictive values. The positive predictive value for delivery within 24 h was 43.9%, with a sensitivity of 72.3% and specificity of 69.0%, when sFlt−1 was <8,682 pg/ml. A sFlt-1 level of 7,394 pg/ml or greater would result in delivery within 1 week, with a positive predictive value of 67.2%; the sensitivity was 79.0% and specificity was 72.0%.

**Conclusion:**

This study showed that sFlt-1 may be effective in predicting the timing of pregnancy termination. However, the number of cases was small and, thus, the results were not definitive. This finding should be researched further in order to predict the optimal timing of pregnancy termination in PE to reduce severe maternal complications.

## Introduction

Preeclampsia (PE) is a disease with serious consequences for both the mother and fetus. Currently, deaths due to complications from PE are problematic in Japan (1–4). In particular, cerebral hemorrhage is more prevalent than cerebral arteriovenous malformation or Moyamoya disease in patients with PE (1–4). We have previously analyzed cases of PE-associated cerebral hemorrhage in Japan; the prognosis of PE-associated cerebral hemorrhage is determined by the degree of cerebral hemorrhage. Therefore, a backward analysis of those cases concluded that it was difficult to save a patient's life when cerebral hemorrhage was severe (5). Intervention before the onset of cerebral hemorrhage is a means of reducing maternal death from cerebral hemorrhage complicated by PE. Furthermore, Japanese people including to pregnant women are reported to be more prone to cerebral hemorrhage than cerebral infarction (4).

Once PE develops, it progressively worsens; there is currently no treatment other than terminating the pregnancy once PE becomes severe (6–8). Pregnancy cannot be easily terminated at <37 weeks due to fetal prematurity. Once PE develops, various clinical parameters (blood pressure, proteinuria, et al.) that indicate PE deterioration should be carefully monitored. The pregnancy is terminated when PE is determined to have become severe. However, this intervention method does not eliminate the mother's risk of developing cerebral hemorrhage. Furthermore, because the incidence of cerebral hemorrhage itself is very low, it is difficult to design studies to predict cerebral hemorrhage.

It is widely reported that placental growth factor (PlGF), an angiogenic factor involved in placentation, and its inhibitor, soluble fms-like tyrosine kinase-1 (sFlt-1), are involved in the pathogenesis of PE. The sFlt-1/PlGF ratio has attracted attention as an indicator to predict the onset of PE (9, 10). We hypothesized that PlGF and sFlt-1 could be used to predict the timing of PE pregnancy termination in patients who develop PE. Predicting the timing of PE pregnancy termination would allow for earlier intervention in PE cases. Therefore, the purpose of this study was to investigate the use of PlGF and sFlt-1 to predict the timing of pregnancy termination in women with known PE onset.

## Methods

### Study Population

This study included pregnant women diagnosed with PE (22 weeks 0 days to 33 weeks 6 days gestation) at Mie University Hospital from 1 January 2016, to 30 October 2021, whose pregnancies were terminated for maternal indications (severe PE). Cases in which PlGF and sFlt-1 were measured at the time of diagnosis were enrolled.

### Study Design

This was a single-center observational study. The study design was approved by the Ethics Committee of Mie University Hospital (approval No. H2022-013).

The levels of sFlt-1 and PlGF, and the sFlt-1/PlGF ratio were examined to determine which marker most significantly correlated with predicting the severity of PE after PE onset. If preterm delivery is anticipated in clinical practice, steroids should be administered to protect the fetus. Therefore, it is important to predict delivery within 24 h and 1 week in terms of onset and duration of steroid effects. The first group was defined as having delivered at less than 24 h (T group) and the second group as having delivered at more than 24 h (P group). Second, the time from measurement to pregnancy termination near to delivery was divided into two groups: <1 week (T group) and more than 1 week (P group). For the markers that were best correlated, cutoff values were calculated to predict pregnancy termination at <24 h and <1 week.

### Diagnostic Criteria for PE

Diagnostic criteria for PE-related disorders were based on the International Society for the Study of Hypertension in Pregnancy (ISSHP) guidelines (11), which are as follows: after 20 weeks of gestation, hypertension (systolic blood pressure >140 mmHg, diastolic blood pressure >90 mmHg, or both) and proteinuria (>2+ protein on dipstick urine test, >300 mg protein per 24-h urine collection, >30 mg/dl protein on a spot urine sample, or a protein to creatinine ratio of >30 mg/mM), both of which are defined as new onset (11).

### Criteria for Pregnancy Termination

After the diagnosis of PE, the decision to terminate the pregnancy was made if PE became severe. Severe disease was defined as meeting one of the following criteria: persistent hypertension (systolic blood pressure >160 mmHg or diastolic blood pressure >110 mmHg); Hemolysis, Elevated liver enzymes, and Low Platelets (HELLP) syndrome; renal dysfunction (serum creatinine >1.0 mg/dl or oliguria); thrombocytopenia (<100,000/μl); eclampsia; pulmonary edema; or stroke.

Pregnancies were also terminated in case of fetal growth retardation, even if the patient was judged to have a carefully controlled non-reassuring fetal status.

### Evaluation of Serum Markers

Serum samples (≥2 ml) collected according to standard operating procedures were retrospectively analyzed in the Obstetrics and Gynecology Laboratory of Mie University. Concentrations of sFlt-1 and PlGF in maternal serum (both measured in pg/ml) were determined using an electrochemiluminescence immunoassay platform (Cobas E Analyzer, Roche Diagnostics) with a fully automated Elecsys assay for sFlt-1 and PlGF. The data obtained from the assay were then used to calculate the sFlt-1/PlGF ratio. The within-run coefficient of variation for control samples was <4% for both assays. Between-run coefficients of variation ranged from 2.3 to 5.6% for the Elecsys sFlt-1 assay and 2.4 to 4.6% for the Elecsys PlGF assay.

### Statistical Analysis

Descriptive statistics (mean, standard deviation) were calculated for each group, and inter-group comparisons were made using the two-sided Student's *t*-test at a significance level of 5%. The cutoff value for predicting that a pregnancy will terminate within 24 h and <1 week was the value with the highest Youden's index.

## Results

### Maternal Background

Forty-four patients were enrolled in the study. The patient background data is shown in Table 1. The mean age was 34.3 ± 5.6 years. The mean number of weeks for PE onset was 30.5 ± 3.7 weeks, and the mean number of weeks for pregnancy termination was 32.9 ± 4.3 weeks. The method of delivery was cesarean section in 81.1% of cases. The complication rate of fetal growth retardation was 22.7%, and the mean birth weight was 1759.6 ± 757.1 g.

**Table 1:** Maternal and neonatal background.

	***n* = 44**
Age (years)	34.3 ± 5.6
Hight (cm)	158.6 ± 5.4
Weight (g)	58.8 ± 12.9
Primipara	22 (50.0%)
Gestational age at onset preeclampsia (weeks)	30.5 ± 3.7
Gestational age at delivery (weeks)	32.9 ± 4.3
Delivery mode	
Caesarian delivery	36 (81.8%)
Vaginal delivery	8 (18.2%)
Obstetrics complication	
Fetal growth restriction	10 (22.7%)
Gestational diabetes mellitus	4 (9.0%)
Placental abruption	1 (2.3%)
Birth weight (g)	1759.6 ± 757.1
Z-score of birth weight	−1.1 ± 1.2
UA pH < 7.10	2 (4.5%)
Apgar score (5 mins) <7	5 (11.3%)

### Delivery in Less Than 24 h

**Figure 1:**
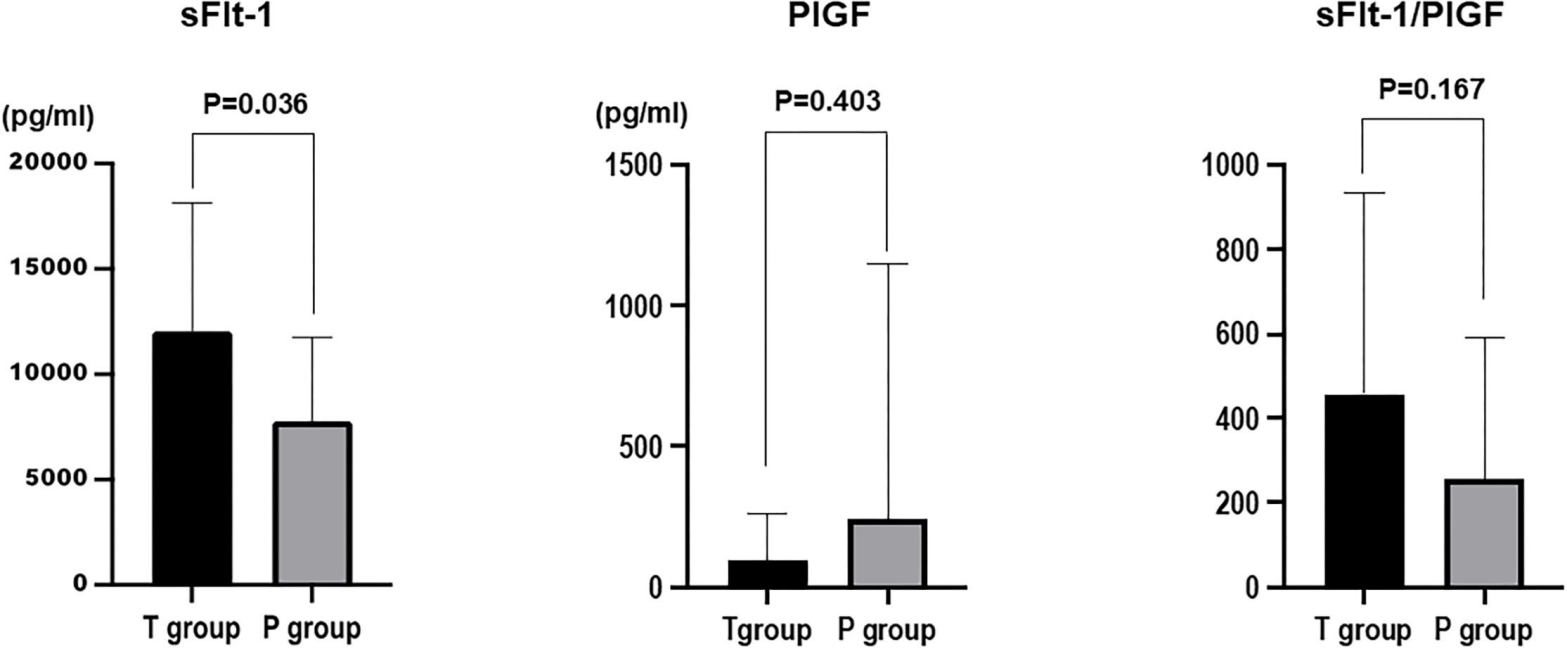
Comparison of sFlt-1, PlGF, and sFlt-1/PlGF ratios between the group that delivered in <24 h (T group) and the group that delivered in more than 24 h (P group).

The mean value of sFlt-1 was 12,034.3 ± 1,379.6 pg/ml in the 24 h T group and 7,779.2 ± 796.5 pg/ml in the 24 h P group (*P* = 0.01). The mean sFlt-1/PlGF ratio was 458.8 ± 112.8 in the 24 h T group and 265.12 ± 66.1 in the 24 h P group (*P* = 0.12). sFlt-1 was the most significantly associated factor with delivery in <24 h (Figure 1).

### Delivery in Less Than 1 Week

**Figure 2:**
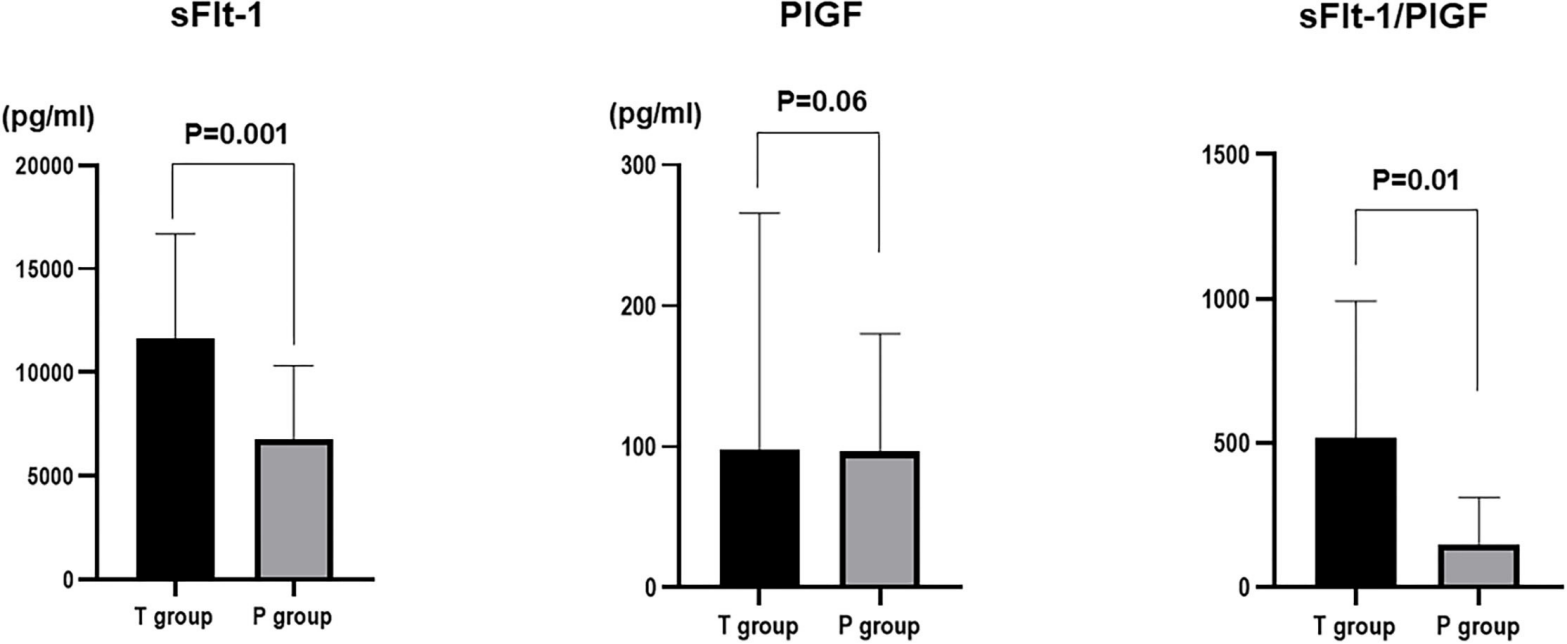
Comparison of sFlt-1, PlGF, and sFlt-1/PlGF ratios in the group that delivered in <1 week (T group) and the group that delivered in more than 1 week (P group).

The mean value of sFlt-1 was 1,1607.5 ± 984.2 pg/ml in the 1-week T group and 6,741.0 ± 858.0 pg/ml in the 1-week P group (*P* = 0.0006). The mean sFlt-1/PlGF ratio was 515.3 ± 76.7 in the 1-week T group and 149.6 ± 66.9 in the 1-week P group (*P*=0.0009). sFlt-1 was the most significantly associated factor with delivery in <1 week (Figure 2).

### Cutoff Values for Delivery in <24 h and <1 Week

**Figure 3:**
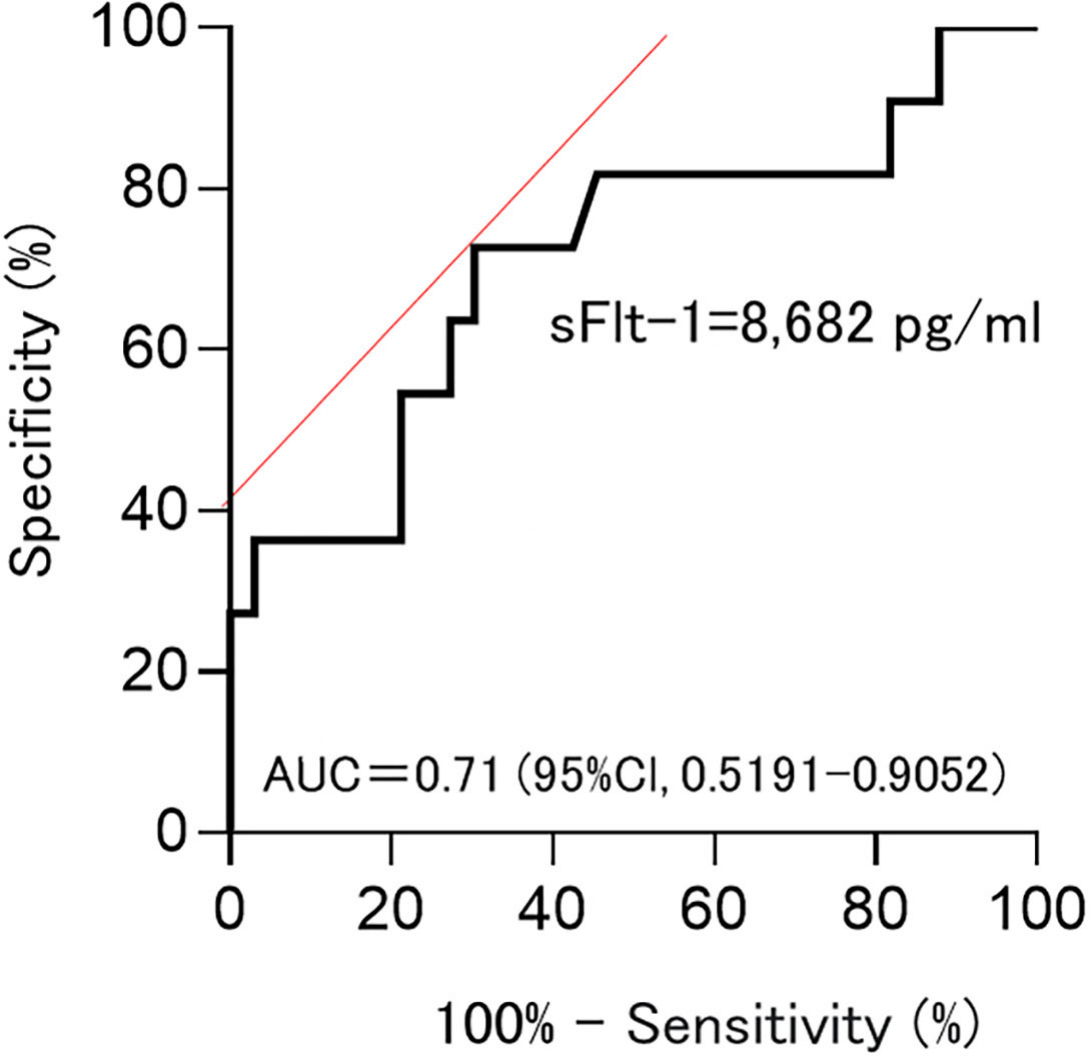
Cutoff values for delivery in <24 h; Cutoff values of 8,682.1 pg/ml (AUC 0.71; 95%Cl, 0.5191–0.9052).

**Figure 4:**
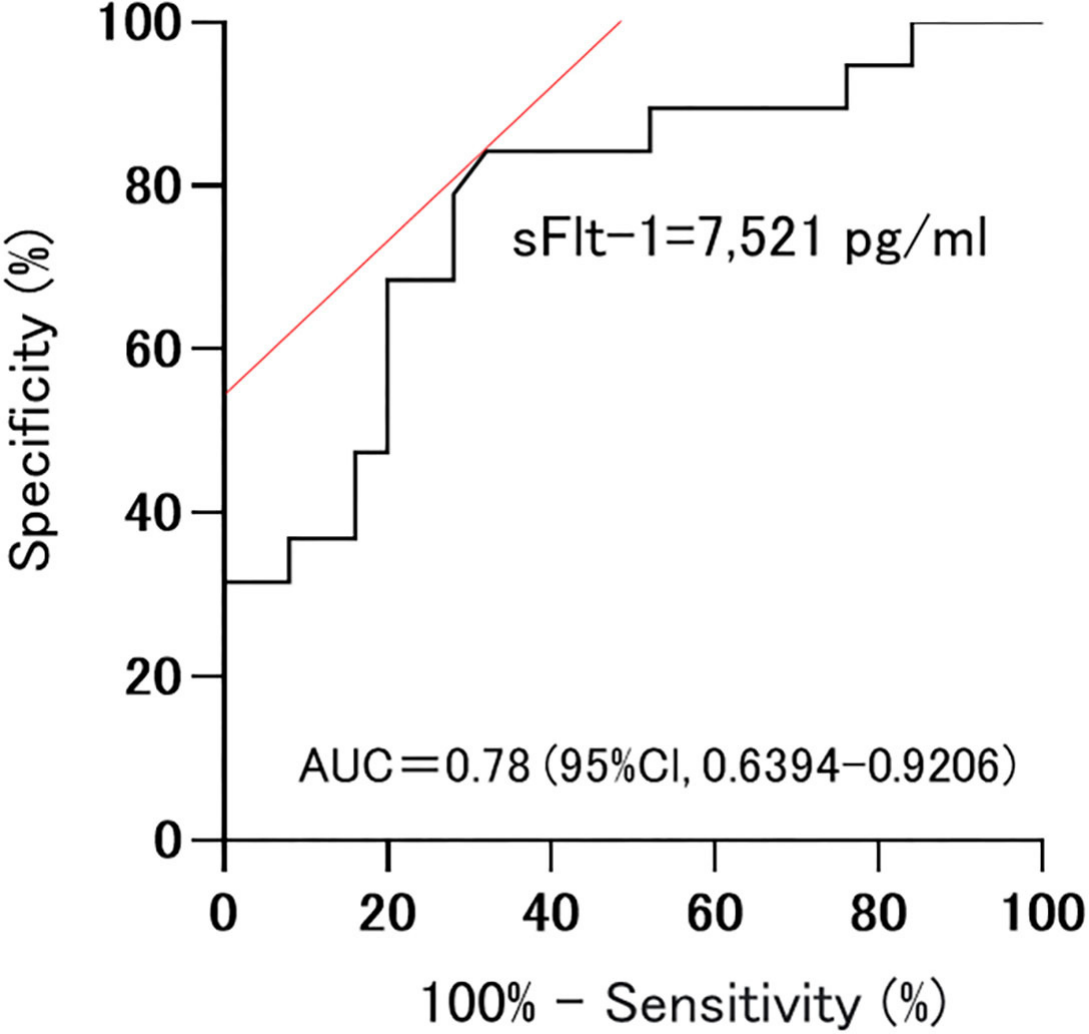
Cutoff values for delivery in <1 week; 7,394.5 pg/ml (AUC 0.78; 0.78, 95%Cl, 0.6394–0.9206).

Among sFlt-1, PlGF, and sFlt-1/PlGF, sFlt-1 was the parameter that was the strongest statistical predictor. Therefore, we used sFlt-1 as the predictor. In this study, cutoff values of 8,682.1 pg/ml (AUC 0.71; 95%Cl, 0.5191–0.9052) and 7,394.5 pg/ml (AUC 0.78; 0.78, 95%Cl, 0.6394–0.9206) for sFlt-1 for delivery in <24 h and within 1 week, respectively, were identified as important predictive values (Figures 3, 4). sFlt-1 below 8,682 pg/ml had a positive predictive value of 43.9% for delivery in <24 h, with a sensitivity of 72.3% and specificity of 69.0%. sFlt-1 >7,394 pg/ml would result in delivery within a week, with a positive predictive value of 67.2%, sensitivity of 79.0%, and specificity of 72.0%.

## Discussion

This study highlights two key novel findings. First, sFlt-1 may be more effective than PlGF or the sFlt-1/PlGF ratio in predicting the time of termination in pregnant women with known PE. Second, sFlt-1 cutoff values were calculated to predict the time of termination of pregnancy (<24 h and <1 week) after PE.

Although the etiology of PE remains controversial, the discovery of cardiovascular angiogenic factors has led to significant advances in both the diagnosis and prediction of prognosis. Of particular interest are the anti-angiogenic factor sFlt-1 and angiogenic factor PlGF. The sFlt-1 level increases in maternal blood in PE, whereas the PlGF level decreases as impaired spiral artery remodeling worsens placental return (12, 13). Therefore, various clinical studies have been conducted using the sFlt-1/PlGF ratio. The sFlt-1/PlGF ratio can be used to predict the development of PE in pregnant women who may be at risk of PE, and for the management planning and decision making in pregnant women with clinical features that are suspicious of PE or those diagnosed with PE. In particular, the sFlt-1/PlGF ratio is extremely useful in predicting the development of PE in pregnant women with suspected PE (9, 14, 15). In Japan, even standard low-risk pregnant women visit the clinic every 1–2 weeks for a checkup. Therefore, pregnant women with PE can be diagnosed at a relatively early stage. In Japan, after PE is diagnosed, the patient is generally hospitalized for observation. For these reasons, we are more interested in the application of the sFlt-1/PlGF ratio to the management and decision-making of pregnant women with known PE than in predicting the onset of PE. Specifically, we are interested in the possibility of terminating pregnancies after the onset of PE and before serious complications develop.

The association between angiogenic factors and adverse events in pregnant women with suspected PE was studied by Rana et al. (16). These studies could reduce unnecessary hospitalizations and inappropriate discharge of pregnant women with suspected PE and reduce the considerable morbidity associated with unplanned preterm delivery. However, it is not currently possible to determine when a pregnancy should be terminated in pregnant women with known PE.

In the Hypertension and Preeclampsia Intervention Trial At near Term-I (HYPITAT I) study in the Netherlands, it was reported that women with gestational hypertension and mild hypertension should be induced to deliver after 37 weeks gestation, although certain angiogenic factors had not been investigated (17). In a study by Verlohren et al. it was possible to identify pregnant women at risk for impending delivery with different hypertension, chronic hypertension, and gestational hypertension (18). However, it was not possible to determine whether induction of labor should be recommended for these pregnant women.

The sFlt-1/PlGF ratio can be used to predict maternal adverse events in pregnant women with known PE. The higher the ratio, the higher the risk of maternal complications requiring hospital intervention, such as acute pulmonary edema, HELLP syndrome, placental abruption, renal failure, refractory hypertension, and eclampsia (10, 16, 18–22). However, these studies have not indicated when the pregnancy should be terminated.

Recent data has shown that the use of an algorithm based on PlGF levels in women with late preterm eclampsia results in a lower rate of progression to severe PE, fewer maternal complications, and no worsening of the neonatal outcome (23). Similarly, the use of the sFlt-1/PlGF ratio in standard practice has been reported to improve clinical accuracy and correctly identify both at-risk women and at-risk babies (24). In the future, it will be necessary to combine this with neonatal conversion to find the appropriate time to terminate the pregnancy.

Predicting the duration of pregnancy after the onset of gestational hypertension nephropathy would allow the obstetrician not to miss the timing of administering steroids before delivery, the obstetrician to observe the patient more intensively as the pregnancy is predicted to be nearing its end, and the neonatologist to be better prepared for the delivery. On the other hand, there is concern that setting a cutoff value may lead to an increase in excessive preterm births beyond the cutoff value. At this time, the indicator of pregnancy termination should be based on clinical findings.

This study had some potential limitations. Although the focus of this study was on the timing of pregnancy termination, it is necessary to examine this issue from the perspective of neonatal prognosis. However, this study was not conducted from the perspective of neonatal prognosis. Another limitation is the retrospective study design and the small number of cases, even though the cases were managed in a uniform manner. An important limitation of this study is the small number of cases. Though this study themselves show that there are different predictive values, the small group of cases considered may be the reason for this result. In the future, more cases should be added to validate the results of this study. sFlt-1 increases modestly with weeks. However, this study did not adjust for this increase, so caution should be exercised in interpreting the cutoff values in this study.

## Conclusion

In summary, this study showed that sFlt-1 may be effective in predicting the timing of pregnancy termination. However, the number of cases was small and the results were therefore not definitive. In combination with previous reports, we would like to develop this study further to predict the optimal timing of pregnancy termination to prevent cerebral hemorrhage after PE.

## Author's Note

The authors of this article are board-certified Obstetrics and Gynecology physicians at Mie University Hospital, which is the largest Obstetrics and Gynecology resident training institution in Japan. The corresponding author is a member of the Japan Maternal Death Exploratory Committee.

## Data Availability Statement

The original contributions presented in the study are included in the article/Supplementary Material, further inquiries can be directed to the corresponding author/s.

## Ethics Statement

The studies involving human participants were reviewed and approved by Mie University Hospital Medical Review Board. Written informed consent for participation was not required for this study in accordance with the national legislation and the institutional requirements.

## Author Contributions

HT conceived and designed the study, performed data acquisition, and conducted data analysis and interpretation. KT provided statistical expertise. HT wrote the manuscript draft. ST, NE, and TI made major revisions to the manuscript. All authors contributed to the article and approved the submitted version.

## Conflict of Interest

The authors declare that the research was conducted in the absence of any commercial or financial relationships that could be construed as a potential conflict of interest.

## Publisher's Note

All claims expressed in this article are solely those of the authors and do not necessarily represent those of their affiliated organizations, or those of the publisher, the editors and the reviewers. Any product that may be evaluated in this article, or claim that may be made by its manufacturer, is not guaranteed or endorsed by the publisher.
